# Supplementary figures and images for: Tibial cortex transverse transport potentiates diabetic wound healing via activation of SDF-1/CXCR4 signaling
Source: PeerJ. 2023 Sep 15;11:e15894. doi: 10.7717/peerj.15894 (PMC10506586; doi:10.7717/peerj.15894)

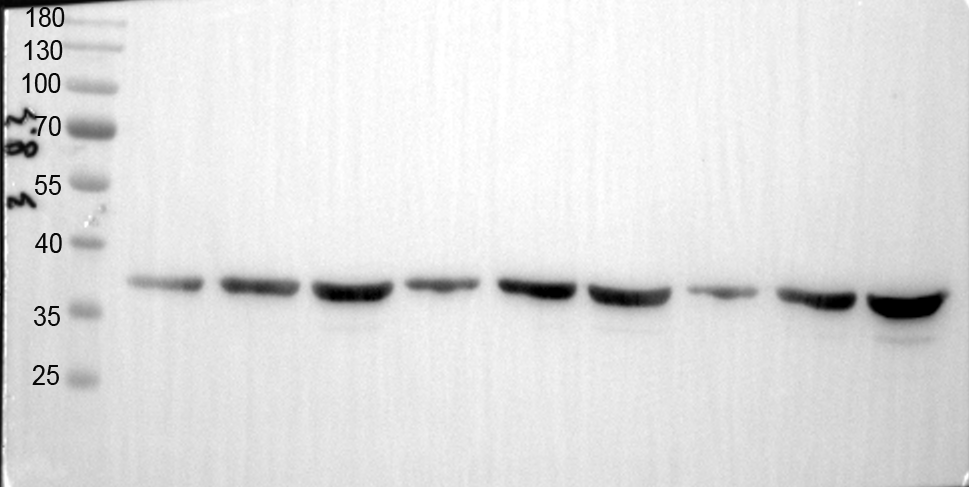

Supplement: Supplemental Information 2 [file peerj-11-15894-s002.zip › full-length uncropped blots/7E. CXCR4.tif]

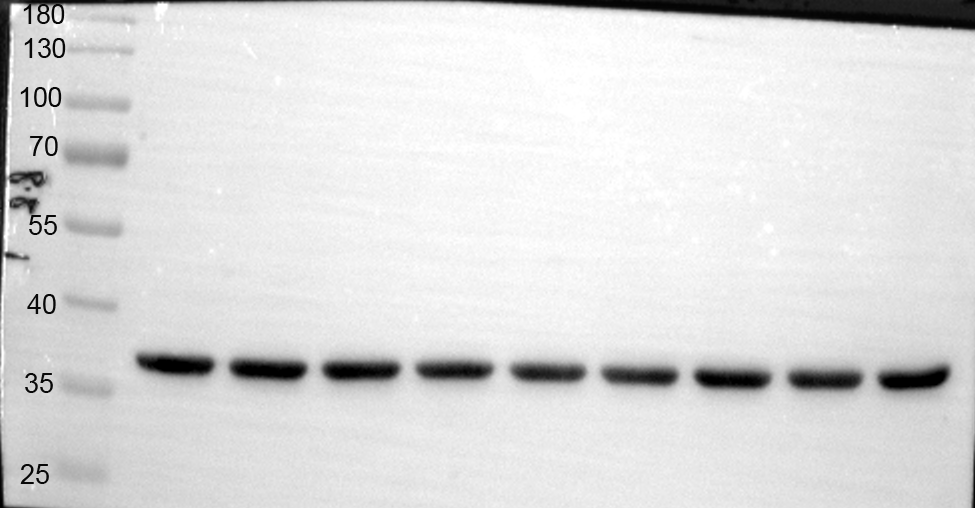

Supplement: Supplemental Information 2 [file peerj-11-15894-s002.zip › full-length uncropped blots/7E. GAPDH.tif]

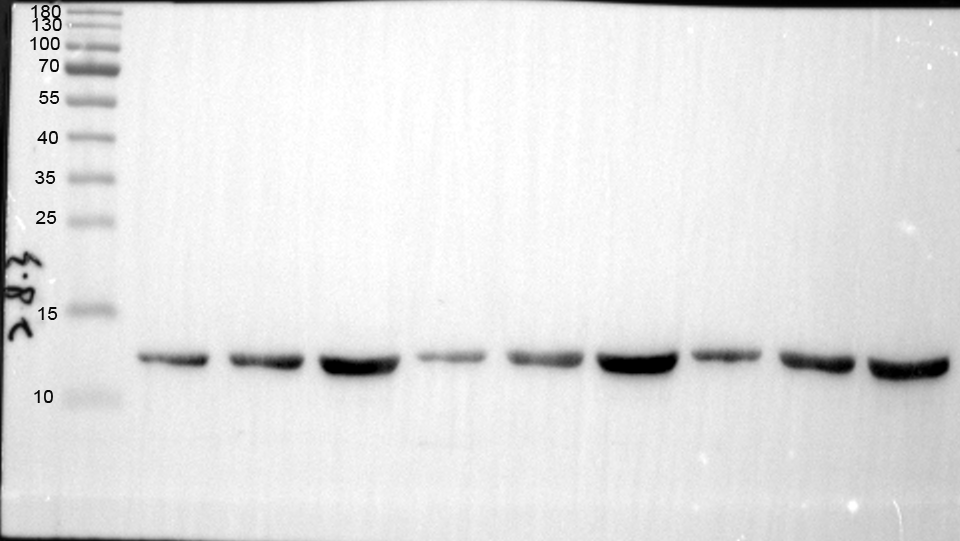

Supplement: Supplemental Information 2 [file peerj-11-15894-s002.zip › full-length uncropped blots/7E. SDF1.tif]
